# Hemorrhagic Shock After Endoscopic Biopsy of Sigmoid Cancer: Pseudoaneurysm of the Rectal Superior Artery

**DOI:** 10.14309/crj.0000000000001074

**Published:** 2023-06-14

**Authors:** Andreas Probst, Florian Schwarz, Florian Sommer, Mousa Ayoub, Helmut Messmann

**Affiliations:** 1 Department of Gastroenterology, University Hospital of Augsburg, Augsburg, Germany; 2 Department of Diagnostic and Interventional Radiology, University Hospital of Augsburg, Augsburg, Germany; 3 Department of General, Visceral and Transplant Surgery, University of Augsburg, Augsburg, Germany

## CASE REPORT

**Figure:**
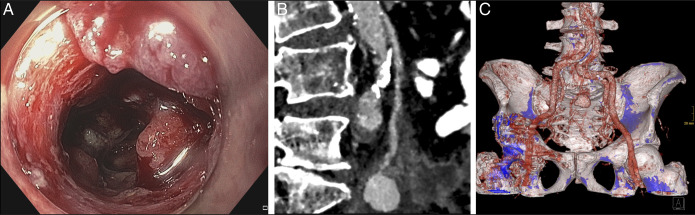

An 86-year-old man without significant comorbidity presented with weight loss, diarrhea, and iron deficiency anemia. No signs of gastrointestinal bleeding were noticed. Colonoscopy was performed, and sigmoid cancer was suspected (**a**). Biopsies were obtained, which confirmed colonic adenocarcinoma. Eight hours after the endoscopy, the patient developed massive hematochezia, followed by hemodynamic instability (blood pressure 60/30 mm Hg; heart rate 120 bpm; and hemoglobin drop from 9.8 g/dL to 6.1 g/dL within 2 hours). After stabilization with intravenous fluids, norepinephrine, and transfusion of 1 unit of red blood cells, endoscopy was repeated and ruled out ongoing bleeding. A computed tomography scan confirmed a mass of the sigmoid colon, which extended along the inferior mesenteric artery and to the os sacrum. Within the sigmoid wall, extravasation of the contrast medium was seen into a structure of 2.3 × 1.7 cm. Computed tomography reconstructions suspected pseudoaneurysm of the inferior mesenteric artery (Figures B, C). In the absence of distant metastases, surgical resection was attempted urgently. However, owing to infiltration of the aortic wall, curative resection was impossible and colostomy was performed. Angiography confirmed a large pseudoaneurysm of the rectal superior artery, and embolization was performed successfully. The further course was unremarkable.

Pseudoaneurysm of the inferior mesenteric artery is rare. To our knowledge, a case with hemorrhagic shock after endoscopic biopsy caused by cancer-associated pseudoaneurysm has not been reported so far.

## DISCLOSURES

Author contributions: A. Probst: conception and design, analysis and interpretation of the data, drafting of the article. M. Ayoub, F. Sommer, and F. Schwarz: analysis and interpretation of the data. H. Messmann: critical revision of the article, final approval of the article.

Financial disclosure: None to report.

Informed consent was obtained for this case report.

